# Validation of the Reference Genes for the Gene Expression Studies in Chicken DT40 Cell Line

**DOI:** 10.3390/genes11040372

**Published:** 2020-03-30

**Authors:** Aleksandra Dunislawska, Anna Slawinska, Maria Siwek

**Affiliations:** Department of Animal Biotechnology and Genetics, University of Science and Technology, 85-084 Bydgoszcz, Poland; slawinska@utp.edu.pl (A.S.); siwek@utp.edu.pl (M.S.)

**Keywords:** housekeeping genes, chicken B cells, in vitro, qPCR, quantitative gene expression

## Abstract

The selection of a suitable reference gene assures a reliable gene expression analysis when using the qPCR method. Normalization of the reaction is based on the basic metabolism genes. These genes show a constant, unregulated expression in all cells and function throughout their lifetime. In the current study, seven reference gene candidates were screened using RT-qPCR, to determine the best-matched pair of reference genes in the chicken DT40 cell line. The DT40 was derived from bursal lymphoma cells that were subjected to RAV-1 bird retroviral infection. It is a simplified in vitro model that allows tracking the direct interaction of stimulants on the lymphoid population and profiling of the hepatocellular B cell transcriptome. The reference gene analysis was carried out using statistical tools integrating four independent methods—geNorm, Best Keeper, NormFinder, delta Ct and RefFinder. Based on the selected reference genes, the relative gene expression analysis was done using the ddCt method. Complete relative gene expression study on a panel of the target genes revealed that proper selection of reference genes depending on the tissue eliminate decreases in data quality. The *SDHA* and *RPL4* genes constitute stable internal controls as reference genes when analyzing gene expression in the DT40 cell line.

## 1. Introduction

Gene expression studies are based on the qualitative determination of the mRNA abundance of a given target gene in total RNA or mRNA. Gene expression studies can be either absolute or relative. The latter method is more frequently used in gene expression studies and requires reference genes which are used as an internal control to normalize the RNA input [1]. Reference genes are constitutive genes, maintaining the basic cellular functions which are essential, regardless of the specificity of the role it plays in the organism. Therefore, from a biological perspective, they are expressed in all cells under normal and experimental conditions. Their expression is also not affected by the type of tissue, stage of the cell cycle, stage of development, or external factors [2].

There is a high probability that, despite all the precautions, various amounts of matrix are present during the RT-qPCR reaction. Therefore, the RT-qPCR results require normalization. The main causes of differences in the amount of genetic material between samples might be due to variable efficiency of reverse transcription and mRNA isolation. Normalization of the reaction is based on the basic metabolism genes (reference genes). They support the basic metabolic functions and encode ubiquitous proteins. The selection of reference genes is relatively large. Commonly used genes are—*UB* (ubiquitin), *G6PDH* (3-phospho-glyceraldehyde dehydrogenase), *B2M* (β2-microglobulin) or *18SRNA/28SRNA*. It has been proved that, unfortunately, these genes do not always show a constant level of expression in all analyzed tissues and cell types. Therefore, they might not be effective for every experimental procedure [3,4]. Thus, the selection of an appropriate internal control should take place each time the tests are carried out, as each experimental model requires a specific reference gene. Literature gaps in the selection of reference genes for chicken cell lines are the main reason for conducting this type of experiment. In order to realize the effective selection of reference genes, it is necessary to carry out real experiments and put forward research hypotheses.

The main goal of the study was to select the optimal RT-qPCR reference genes for the DT40 chicken cell line and apply these genes for the validation of the whole-genome analysis of the chicken expression microarray. The DT40 chicken cell line used for research came from the Collection of Microorganisms and Cell Cultures. The DT40 line was derived from bursal lymphoma cells that were subjected to RAV-1 bird retroviral infection [5,6]. This caused the immortalization of cells and their ability to survive in culture for many weeks. It is a simplified model that also allows tracking the direct interaction of stimulants on the lymphoid population [7]. The in vitro model allowed us to track biochemical pathways in the lymphoid cell line. The intention of this publication is in silico selection of reference genes and their further verification in a biological model.

## 2. Materials and Methods 

### 2.1. Reference Genes Selection

The gene classified as a reference should be characterized by: (1) constant level of expression, regardless of the prevailing experimental conditions, (2) similar number of transcripts in relation to the number of transcripts of the examined genes, (3) different function than those of the target genes. Based on these criteria and literature data, the selection of genes and selection of the most suitable one for further research was made.

A selected panel of seven reference genes used in the current study is presented in Table 1. The oligonucleotide sequences are based on literature sources.

### 2.2. Sample Collection for Analysis 

The DT40 cell line that was used in this experiment was a wild-type DT40 (not recombinant/transfected) and was purchased from a DSMZ collection (DSMZ no.: ACC 636) in the Leibnitz Institute (Brauschweig, Germany). Cells were treated with four stimulatory factors (bioactive substances)—prebiotic (P), and three different synbiotics (S1, S2, S3). Cell culture conditions were reported earlier by Dunislawska et al. [7]. The experiment was carried out in three biological replications. Five technical repetitions were performed in each biological repetition. The total RNA was isolated from DT40 cells using a Universal RNA Purification Kit (EURx, Gdansk, Poland). A qualitative and quantitative assessment of RNA was carried out using 2% agarose gel and spectrophotometer Nanodrop2000 (Thermo Fisher Scientific, Massachusetts, MA, US). Only RNA of the best quality (good integrity and intensity of agarose gel bands) and purity (absorbance ratio A260/A280 between 1.8 and 2) was intended for further analysis. RNA was used as a template for the RT-qPCR reaction. 

### 2.3. RT-qPCR

cDNA for qPCR analysis was synthesized using the Maxima First Strand cDNA Synthesis Kit (Thermo Scientific/Fermentas, Vilnius, Lithuania) for reverse transcription quantitative PCR (RT-qPCR), following the manufacturer’s recommendations. Prior to qPCR amplification, cDNA was diluted to 70 ng/µL. The reaction mixture for the qPCR step included Maxima SYBR Green qPCR Master Mix (Thermo Scientific/Fermentas, Vilnius, Lithuania), 1 μM of each primer, and 2 μL of diluted cDNA (140 ng). Thermal cycling was performed in a LightCycler II 480 (Roche Diagnostics, Basel, Switzerland). The thermal profile of the reaction was as follows—initial denaturation (15 min, 95 °C), 40 amplification cycles which consisted of—denaturation (10 s, 95 °C), primer attachment (15 s, 58 °C) and elongation (30 s, 72 °C). Each RT-qPCR reaction was conducted in two technical replicates. Melting curves were evaluated for each tested gene. Reaction efficiency analysis was also carried out on the basis of the optimization of the reactions and primers. Standard curve analysis was performed to calibrate qPCR and to verify the reaction changes along with matrix concentration and assess the reaction efficiency. A series of 10-fold matrix dilutions were made to generate standard curves. Reaction efficiency was calculated automatically in LightCycler 480 SW 1.5 software during curve generation. RT-qPCR reaction included two gene panels—(1) reference genes and (2) target genes (based on differentially expressed genes in microarray). 

### 2.4. Statistical Tools to Select the Appropriate Reference Genes

In order to select the optimal reference genes, analysis was carried out using statistical tools—geNorm [4], BestKeeper [12], NormFinder [13] and Delta Ct method [14]. Comprehensive stability rankings were obtained with RefFinder [15]. 

The geNorm analysis [4] creates a ranking of reference genes based on the expression stability. According to this method, the expression level ratio of the two optimal reference genes should remain unchanged for all tested samples. The higher the variation in the expression level ratio, the lower the stability of a given gene. The M value is used to determine the stability of a single gene relative to the rest. This is an average of the calculated standard deviations for each pair of genes. The lower the M value, the greater the stability of the gene [9,16]. 

The BestKeeper analysis [17] gives the following parameters—geometric mean (GM), minimal value (Min), maximal value (Max), standard deviation (SD), Pearson coefficient (r). The first step in result interpretation is to determine the stability of the genes based on the SD value. If the SD value for a given gene is greater than 1, it is removed from further analysis. BestKeeper analysis allows for the elimination of genes that significantly deviate from other levels of expression. If any of the analyzed genes is characterized by three times reduced or increased expression compared to the average level of expression of all analyzed genes, its elimination is recommended. BestKeeper also examines the mutual correlations between individual gene pairs. The obtained Pearson coefficient can show a positive correlation (if r = 1), negative (if r = -1) or no correlation (if r = 0) [9].

According to the NormFinder analysis [13], the optimal reference gene is the one with the lowest SV stability (Stability Value). The higher the SV, the greater the variability of gene expression under specific conditions [10,16]. 

The delta Ct method [14] can also be used to select reference genes. It compares the relative expression of "gene pairs" in each sample, which allows the selection of optimal genes for basic metabolism. If the delta Ct between two genes remains constant for the different samples analyzed, it means that both genes are permanently expressed. The introduction of more genes to comparisons provides a larger pool of information whereby pairs exhibit less variability, and thus which genes show stable expression among the tested samples.

The final ranking was built based on RefFinder [15]. RefFinder exploits previously mentioned computational programs using the geometric mean of the attributed weights.

### 2.5. Target Genes

To validate the performance of the reference genes analyzed in this study, we performed a complete relative gene expression study on a panel of the target genes and compared the results to the gene expression microarray data. The panel of the target genes was selected based on the microarray experiment based on the DT40 cell line. DT40 was stimulated in vitro with LPS, prebiotic (P) (RFOs–raffinose family oligosaccharides), and three synbiotics—S1 (RFOs + *Lactococcus lactis* subsp. *lactis* IBB SL1), S2 (RFOs + *Lactococcus lactis* subsp. *cremoris* IBB SC1) and S3 (commercial synbiotics, lyophilized strains of *Lactobacillus acidophilus* and *Streptococcus faecium* with the addition of lactose). The synbiotics mixture was heat-deactivated prior to stimulation (95 °C, 5 min, conditions based on literature). The stimulation was carried out for 9 hours, followed by RNA isolation and NimbleGen Gene Expression Microarray (custom design). There have been one control group and five treatment groups in our data—prebiotics, synbiotics 1 to 3 and LPS. We did two analyses, the difference between them being a normalization method applied to the data. Both analyses were done using the R software (the limma package) and, for all technical repeats, one randomly selected repeat has been randomly removed. The goal was to find genes differentially expressed between each of the treatment groups and the control group. At first, we used data normalized using the rma method. To find the genes of interest, a linear model was built explaining expression of each gene within each group. Expression of genes between each treatment group and controls was compared and, for each comparison, only genes with statistically significant (FDR ≤ 0.05) differential expression and ratio either lower than 0.61 or higher than 1.66 were selected. To be sure that the results we got are not influenced by the specific normalization method, we did a secondary analysis. To this end, we took raw expression values for each probe in two groups—controls and LPS. Three normalization methods were applied to these data—vsn, normalizeBetweenArrays and normalizeBetweenArrays with background correction. Three lists of genes that are differentially expressed between LPS and controls were created, one for each normalization method. These lists were compared against each other and with the results obtained for the rma normalization method (first analysis). Based on this comparison and knowledge about which genes should show up as differentially expressed between LPS and controls, we selected vsn normalization for further use.

Gene selection was based on the criteria—(A) statistical significance of the results obtained and (B) changes in the level of gene expression in stimulated groups. Primers were designed based on following criteria—(1) the optimum melting temperature (Tm) in the temperature range between 58 and 60 °C, (2) one of the designed primers should have interlocking exon-exon regions, (3) the amplicon should be from 50 to 150 bp for optimal PCR performance, (4) the recommended length of the primer is 20 bases, (5) the percentage of GC base pairs should be in the range of 30% to 80% without more than two G + C residues at the 3’ end of the five last nucleotides, and (6) avoiding duplication of identical oligonucleotides. 

### 2.6. Relative Gene Expression and Statistical Analysis

To avoid errors during relative expression analysis, the number of transcript normalization of the test gene against the number of transcripts of the reference gene was used. The comparative method (ddCt) was used for the analysis [18]. For each trial, differences between the Ct values of the test and reference genes were calculated. The following formula was used to calculate the normalized value of the expression level of the test gene in relation to the calibrator, i.e., the control sample—R = 2^−ΔΔCt^. In the last stage, statistical analysis was carried out, using the t-test (* *p* < 0.01; ** *p* < 0.05). Comparison of the fold induction (FC) values for microarrays and qPCR results was the validation of the microarray results.

## 3. Results

### 3.1. Reference Genes Selection 

In our research, seven reference genes were analyzed—*G6PDH, SDHA, B2M, RPL4, UB, HPRT* and *RPL30*. Based on the dCt analysis method, the most stable genes turned out to be *RPL4* and *SDHA*, while the least stable ones were *RPL30* and *B2M*. The BestKeeper method showed that all genes had SD values below 1. The *G6PDH* gene proved to be the most stable, while the RPL30 the least. Combinations of the three best genes, *G6PDH, RPL4* and *SDHA,* had a positive r-value. In the NormFinder algorithm, the *RPL4* and *SDHA* genes had the lowest stability values. The *RPL30* and *B2M* genes turned out to have the highest expression variability (0.15 and 0.162, respectively). After the geNorm analysis, the *SDHA* and *RPL4* genes had the same M value (0.079) and, at the same time, the lowest among all genes. The highest M values were for *RPL30* (0.146) and *B2M* (0.16).

The results of all algorithms were analyzed using the RefFinder tool which integrates four algorithms—geNorm, BestKeeper, Norm Finder and Delta Ct (data presented in Table 2).

**Figure 1:**
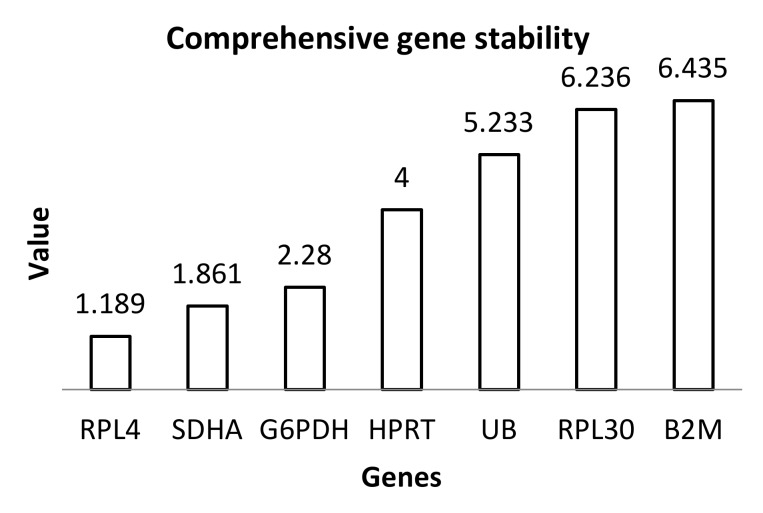
Graph presenting the results of reference gene analysis taking into account all available methods in the RefFinder tool. The value calculated by RefFinder is based on raw Ct values for the input.

Based on the obtained results, the most stable genes turned out to be *RPL4* and *SDHA*, while the least stable ones were *RPL30* and *B2M*. Figure 1 illustrates a summary of the analysis results of all the applied methods available in RefFinder.

### 3.2. Amplification Efficiencies in the qPCR Analysis

**Figure 2:**
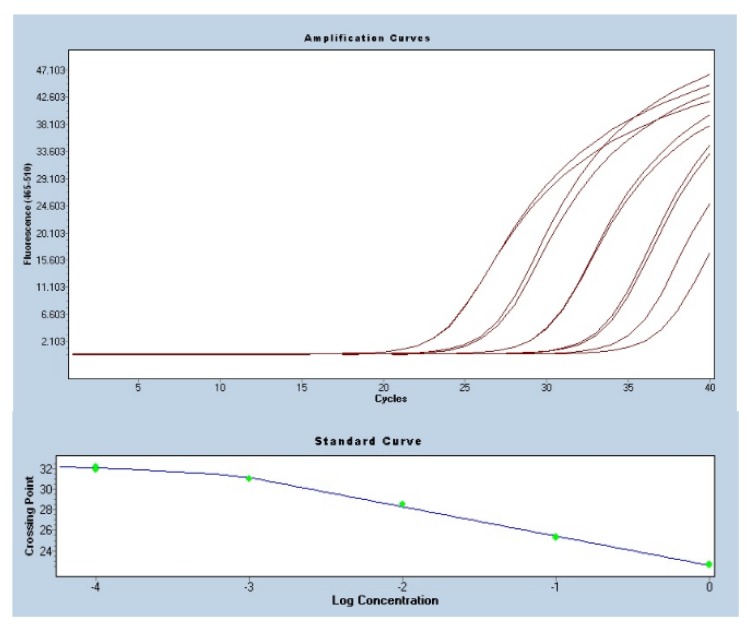
Amplification curves and standard curve obtained for a series of 10-fold dilutions of the cDNA for the *UB* gene. The RT-qPCR reaction used to generate curves was performed in a LightCycler480 (Roche) thermal cycler in two technical replications for one randomly selected sample, with SYBR Green as the intercalating dye.

Analysis of the reaction efficiency was carried out on the basis of standard curves generated based on a series of 10-fold matrix dilutions. The slope of the graph, necessary to determine the reaction efficiency, was determined on the basis of standard curves. The average slope of the curve graph for the analyzed genes was −3.51. The reaction efficiency was 1.97 on average. This result indicates a 98% qPCR efficiency. The sample amplification curves for UB obtained as a result of the qPCR reaction are shown in the Figure 2. The slope of the UB curve was −2.84. The reaction efficiency for UB was 2.25.

### 3.3. Target Genes Selection

The number of differentially expressed genes upon stimulation based on the microarray data is as follows:

LPS: 155 genes => Gene Ontology (GO) indicates immune response (chemotaxis, response to LPS)

P: 667 genes => GO: G-protein signaling pathway, cell surface receptor linked signal transduction, regulation of transcription, embryogenesis)

S1: 49 genes => no GO hits/no response

S2: 637 genes => 397 common genes between P and S2, GO: G-protein signaling pathway, cell surface receptor linked signal transduction, cation transport

S3: 37 genes => no GO hits/no response

A final group of five genes was selected based on higher changes in level of expression and statistical significance. Selected genes with designed primers are presented in Table 3. 

### 3.4. Comparison qPCR Results with Microarrays Data Based on the Selected Reference Genes

For further analyses, the geometric average of the most stable genes, *RPL4* and *SDHA* (as a positive control), and geometric average of the least stable genes, *RPL30* and *B2M* (as a negative control with reference genes shown as the weakest by RefFinder), were used. 

**Figure 3:**
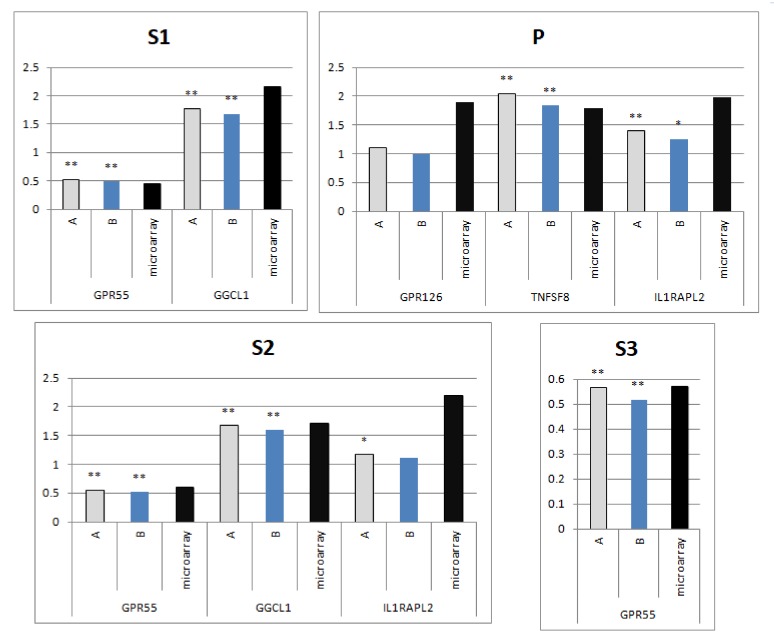
(**A**) The best performing reference genes (*RPL4, SDHA*) and (**B**) the worst performing reference genes (*RPL30, B2M*) used to calculate relative gene expression of tested genes. Comparison of experimental groups with the control group by Student’s t-test; * for *p* <0.05 and ** for *p* <0.01. The y-axis presents the changes in gene expression relative to control calculated by dCt formula (fold induction). The x-asis presents analyzed genes.

The selected genes from the microarray experiment were assigned to four separate experimental groups—P, S1, S2 and S3. Within all experimental groups, gene expression after a relative gene expression analysis based on *SDHA* and *RPL4* (the most stable genes) was closer to the microarray results than in the case of normalization using the *B2M* and *RPL30* genes (least stable genes). Results for all groups of stimuli comparing RT-qPCR results to microarray are presented on Figure 3. 

Analysis of the results in the stimulated groups of panel genes showed an increase in expression of all genes, confirming the results of the microarrays (Figure 3). Almost all genes (except for *GPR126* in the P group) had statistically significant results. The ddCt analysis confirmed the existence of dependence between data from the microarray analysis and RT-qPCR. 

## 4. Discussion

RT-qPCR method is widely used in molecular biology research to estimate gene expression level. Hence, to obtain biologically relevant data, normalization of a stable reference gene is a key factor in experiments. The expression of reference genes should be stable in any experimental conditions and in any type of tissue. However, there is no universal stable gene in each condition and in every tissue. Based on this, the reference gene stability has to be verified for qPCR studies in each type of tissue. In this study we compared reference genes for the DT40 cell line to select appropriate particular ones and combine optimal pairs of reference genes as a key factor in the analysis by the RT-qPCR method. The use of the chicken cell lines in vitro, especially DT40, is increasing due to the ease with which it can be manipulated. The DT40 cell line is an attractive option for the analysis of single and multiple gene expression. This cell line has been used in analyses of the cell cycle regulation, B cell antigen receptor (BCR) signaling, apoptosis or gene conversion, among others [19].

### 4.1. Reference Genes Selection

A reference gene (also known as housekeeping gene) is defined as a gene which is transcribed at a relatively constant level in various conditions, which do not affect its expression. However, stability of all the reference genes is not constant. The most stable gene in one condition or one treatment might be highly variable in another one. Therefore, it is necessary to select proper reference genes dedicated to specific tissues or treatments. We analyzed the data of the DT40 cell line stimulated with different bioactive substances. Our goal was to define the best reference genes for DT40 cell line treatment.

It has been reported that the commonly used genes, e.g., *G6PDH* and *UB,* have a constant expression level in chicken tissues infected with the virus, whereas *HPRT* is an effective reference for several species of poultry, including chickens [9]. Nevertheless, literature reports have shown that all selected reference genes differ depending on the tissue. Therefore, optimization of reference genes or verification of optimal reference genes for a specific tissue is necessary before each experiment [20,21,22]. The impact of experimental conditions on the expression of a single reference gene should be eliminated. 

Our results pointed out two the most stable reference genes—*SDHA* and *RPL4*. SDHA is a gene coding for the main catalytic subunit of succinate-ubquinone oxidoreductase, which is an enzyme of the respiratory chain and the citric acid cycle. The *RPL4* gene is responsible for coding the ribosomal L4 protein, which is a component of the 60S subunit. In our study, the least stable reference genes turned out to be *RPL30* and *B2M*. The choice of a single gene for normalization may involve a relatively large error, which is why the geometric mean of the two best reference genes was used for the analysis. Literature recommend the use of multiple genes geometrically averaged to control for outliers [17]. 

### 4.2. Comparison qPCR Results with Microarrays Data Based on Selected Reference Genes

Both the microarray and qPCR methods require complex normalization procedures. Any differences between RT-qPCR fold induction and microarray fold changes can be explained by differences in coverage with oligonucleotides in these techniques. Microarray changes might be based on gene families with similar fragment sequences connecting the probe. Another reason explaining differences between gene expression changes in both methods may be the fact that microarrays include the entire gene (all exons). Primers for qPCR include on average two exons only [23]. Single gene analysis is more precise, but the analysis of the whole group of genes or whole molecular pathways is more complex and informative and the choice of the method depends on the analyses carried out.

## 5. Conclusions

Reference genes have to be selected with great care to eliminate drastic decreases in data quality due to badly matched genes. The RT-qPCR reaction proved to be an effective method of validating the results of the microarray experiment. The *SDHA* and *RPL4* genes constitute stable internal controls as reference genes when analyzing gene expression in the DT40 cell line. 

## Figures and Tables

**Table 1:** Sequences of the reference gene panel.

Gene	Gene name	Primer sequence (5′-3′)	Reference
**G6PDH**	glucose-6-phosphate dehydrogenase	F: CGGGAACCAAATGCACTTCGT R: GGCTGCCGTAGAGGTATGGGA	[8]
**HPRT**	hypoxanthine phophoribosyl-transferase	F: CCCAAACATTATGCAGACGA R: TGTCCTGTCCATGATGAGC	[9]
**SDHA**	succinate dehydro-gense complex, subunit A	F: AGAGCCTCAAGTTCGGGAAG R: CAGGAGATCCAAGGCAAAAT	[10]
**B2M**	beta-2-microglobulin	F: ACTTTTCACACCGCTCCAGT R: CGGATGGAACCCAGATACAT	[10]
**RPL4**	ribosomal protein L4	F: AGGAGGCTGTTCTGCTTCTG R: TCCAGGGATGTTTCTGAAGG	[10]
**RPL30**	ribosomal protein L30	F: GAGTCACCTGGGTCAATAA R: CCAACAACTGTCCTGCTTT	[11]
**UB**	Ubiquitin C	F: GGGATGCAGATCTTCGTGAAA R: CTTGCCAGCAAAGATCAACCTT	[9]

**Table 2:** Results of reference genes analysis—Delta Ct, BestKeeper, NormFinder and geNorm.

Reference Gene	Delta Ct	BestKeeper	NormFinder	geNorm
	Average of STED	Standard Deviation SD	Stability Value	Stability Value
G6PDH	0.146	0.184	0.091	0.093
HPRT	0.166	0.231	0.126	0.11
SDHA	0.132	0.197	0.063	0.079
B2M	0.193	0.241	0.162	0.16
RPL4	0.126	0.194	0.033	0.079
RPL30	0.184	0.264	0.15	0.146
UB	0.17	0.245	0.131	0.13

**Table 3:** Gene panel selected for validation of the microarray experiment using the RT-qPCR method. Gene selection was done based on the microarray experiment.

Gene	Gene Name	Primer Sequence (5´-3´)	Gene ID
**GPR55**	G protein-coupled receptor 55	F: GGAGTCACCCGCTGGAGAA R: TCTCCCCCTGCTGAGATGTT	770641
**GPR126**	G protein-coupled receptor 126	F: TGTGCGAATTGCCGTGTCT R: CACTGCTGCTCTTGGTTGCTT	421673
**TNFSF8**	tumor necrosis factor (ligand) superfamily, member 8	F: GCAAAGGGAACACCTCTGAAGA R: TGAGTTTGACACTCTCATGTATGCA	378930
**GGCL1**	chemokine-like ligand 1	F: TGTATGAGTACACGGGAAGCAGAT R: ACAGCACTTCTTCCCCTCAAAG	417464
**IL1RAPL2**	interleukin 1 receptor accessory protein-like 2	F: GATCTGGCTAACTATACGTGTCATGTG R: ATCAAATCTTTCTTGCGCAGAAG	422379
